# Phase 1/2 trial of ixazomib, cyclophosphamide and dexamethasone in patients with previously untreated symptomatic multiple myeloma

**DOI:** 10.1038/s41408-018-0106-3

**Published:** 2018-07-30

**Authors:** Shaji K. Kumar, Francis K. Buadi, Betsy LaPlant, Alese Halvorson, Nelson Leung, Prashant Kapoor, David Dingli, Morie A. Gertz, Ronald S. Go, P. Leif Bergsagel, Yi Lin, Angela Dispenzieri, Yi Lisa Hwa, Amie Fonder, Miriam Hobbs, Rafael Fonseca, Suzanne R. Hayman, A. Keith Stewart, John A. Lust, Joseph Mikhael, Wilson Gonsalves, Craig Reeder, Tomas Skacel, S. Vincent Rajkumar, Martha Q. Lacy

**Affiliations:** 1 Division of Hematology, Mayo Clinic, Rochester, MN USA; 2 Department of Biostatistics, Mayo Clinic, Rochester, MN USA; 3 Division of Hematology and Oncology, Mayo Clinic, Phoenix, AZ USA; 4 Takeda Pharmaceutical Company Limited, Osaka, Japan; 5 31st Medical Department Clinical Department of Haematology of the First Faculty of Medicine and General, Teaching Hospital Charles University, Praha, Czech Republic; 6 Clinical Department of Haematology of the First Faculty of Medicine and General Teaching Hospital, Charles University, Praha, Czech Republic

## Abstract

Ixazomib is the first oral proteasome inhibitor to enter the clinic. Given the efficacy of bortezomib in combination with cyclophosphamide and dexamethasone, we studied the combination of ixazomib, cyclophosphamide and dexamethasone (ICd) in newly diagnosed multiple myeloma (NDMM) and patients with measurable disease, irrespective of transplant eligibility, were enrolled. The phase 1 was to determine the maximum tolerated dose (MTD) of cyclophosphamide in the combination. Patients received ixazomib 4 mg (days 1, 8, 15), dexamethasone 40 mg (days 1, 8, 15, 22), and cyclophosphamide 300 or 400 mg/m^2^ days 1, 8, 15, 22; cycles were 28 days. We enrolled 51 patients, 10 in phase 1 and 41 patients in phase 2. The median age was 64.5 years (range: 41–88); 29% had high or intermediate risk FISH. The MTD was 400 mg/m^2^ of cyclophosphamide weekly. The best confirmed response in all 48 patients included ≥ partial response in 77%, including ≥ VGPR in 35%; 3 patients had a sCR. The response rate for all 48 evaluable patients at 4-cycles was 71%; the median time to response was 1.9 months. Common adverse events included cytopenias, fatigue and GI intolerance. ICd is a convenient, all oral combination that is well tolerated and effective in NDMM.

## Introduction

The initial treatment of multiple myeloma has undergone a dramatic change in the past decade with the routine incorporation of the proteasome inhibitors and/or immunomodulatory drugs (IMiDs) in induction regimens^1,2^. Multiple phase three trials have confirmed the beneficial role of these drugs for the initial treatment of myeloma, with longer duration of disease control as well as improved overall survival^3,4^. Proteasome inhibitors in particular have become an integral part of the upfront regimens for MM, and appear to have significant impact on the outcome in patients with certain cytogenetic abnormalities associated with aggressive disease behavior^5,6^. Proteasome inhibitors, when combined with immunomodulatory drugs such as lenalidomide or alkylating agents, have resulted in some of the most effective treatment regimens in myeloma to date^7–9^. Bortezomib, the initial proteasome inhibitor to enter the clinic, has been combined with a variety of different drugs^10–13^. In a meta-analysis of randomized clinical trials of initial therapy of myeloma, the use of bortezomib was associated with better overall survival^14^. Bortezomib, in combination with alkylating drugs such as melphalan and cyclophosphamide, is used extensively in patients with newly diagnosed myeloma^15–17^. The combination of bortezomib, cyclophosphamide and dexamethasone (VCD) has been studied in several phase 2 studies as well as a recent phase 3 trial, where it was compared with bortezomib, thalidomide and dexamethasone (VTD)^16,18–20^. VCD is an effective combination, allowing rapid and deep control of the disease in previously untreated myeloma. However, it is associated with high risk of peripheral neuropathy and also requires weekly clinic visits for parenteral administration. While the risk of peripheral neuropathy with bortezomib has been mitigated to some extent with the weekly schedule and the use of subcutaneous administration, it still remains of concern^21,22^.

Ixazomib citrate (MLN9708) is an investigational inhibitor of the 20S proteasome that represents the first orally bioavailable proteasome inhibitor to be evaluated for treatment of MM^23^. Ixazomib citrate is a modified peptide boronic acid and is the citrate ester of ixazomib (MLN2238), the biologically active moiety. Ixazomib citrate rapidly hydrolyzes to ixazomib upon contact with aqueous solution or plasma. Ixazomib preferentially binds the β_5_ site of the 20S proteasome at lower doses, with inhibition of the β_1_ and β_2_ sites at higher concentrations. Compared to bortezomib, nonclinical studies have shown that ixazomib has a faster dissociation rate from the proteasome. Ixazomib has demonstrated antitumor activity in a range of tumor xenograft models, including MM models^24,25^. In clinical trials, ixazomib has shown promising activity as a single agent in patients with relapsed and refractory MM, with very low rates of peripheral neuropathy observed in single agent trials^15,26,27^. Ixazomib has shown considerable efficacy in combination with immunomodulatory drugs in newly diagnosed and relapsed, refractory myeloma^15,26,28–30^. It is approved is over 50 countries for use in relapsed myeloma, based on the results of a phase 3 trial demonstrating improved PFS when added to lenalidomide and dexamethasone. Given the significant clinical activity of the VCD regimen in previously untreated MM, we wanted to explore the efficacy of replacing bortezomib with ixazomib in combination with cyclophosphamide and dexamethasone.

## Patients and methods

### Study design

This was a sequential phase 1 study designed to determine the optimal dose of cyclophosphamide to be combined with ixazomib and dexamethasone followed by a phase 2 component to evaluate the safety, tolerability and efficacy of combining weekly oral cyclophosphamide with oral ixazomib citrate given weekly along with dexamethasone in patients with previously untreated MM (NDMM). The phase 1 portion utilized the standard cohort 3 + 3 design. Three patients were treated at each dose level and observed for a minimum of 4 weeks (i.e., one full cycle) before new patients were enrolled into the same or next dose level. MTD was defined as the dose level below the lowest dose that induces dose-limiting toxicity (DLT) in at least one-third of patients (at least 2 of a maximum of 6 patients). Doses were not escalated in any individual patient. The study enrolled 51 patients between August 2013 and August 2015. The study was performed in accordance with the provisions of the Declaration of Helsinki, the International Conference on Harmonization, and the Guidelines for Good Clinical Practice, and with approval of the Mayo Clinic Institutional Review Board. The study was registered at www.clinicaltrials.gov as NCT01864018.

### Study objectives

The primary objective of the phase 1 portion of the study was to determine the maximum tolerated dose of cyclophosphamide that can be combined with ixazomib and dexamethasone in patients with previously untreated symptomatic MM. The primary objective of the phase 2 portion was to determine the complete (CR) plus very good partial response (VGPR) rate (≥VGPR) of ixazomib, used in combination with cyclophosphamide and dexamethasone in patients with previously untreated symptomatic MM. Secondary objectives included an assessment of progression-free survival and overall survival among patients with previously untreated symptomatic MM following treatment with ixazomib in combination with cyclophosphamide and dexamethasone followed by ixazomib maintenance until progression and to determine the toxicities associated with ixazomib used in combination with cyclophosphamide and dexamethasone in patients with NDMM. Correlative studies included assessment of ixazomib pharmacokinetics when combined with cyclophosphamide and dexamethasone and serial evaluation of neurotoxicity using patient completed questionnaires.

### Patient selection

The study enrolled patients, 18 years of age or older, with newly diagnosed MM fulfilling the IMWG criteria for symptomatic MM and who have not received any prior therapy for myeloma. Patients were required to have measurable disease (serum M-protein ≥ 1 g/dL or urine M-protein ≥ 200 mg/24 h or involved free light chain level ≥ 10 mg/dL provided the serum free light chain ratio was abnormal), Eastern Cooperative Oncology Group performance status of 0–2, adequate hematologic (absolute neutrophil count ≥ 1000/mm^3^, platelets ≥ 75,000/mm^3^), hepatic (total bilirubin ≤ 1.5 × upper limit of normal [ULN], alanine/aspartate aminotransferase ≤ 3 × ULN), and renal (creatinine clearance ≥ 30 mL/min) function. Patients with grade ≥ 3 peripheral neuropathy or grade 2 with pain, grade > 1 diarrhea, or who had major surgery or serious infection within 14 days prior to start of therapy were excluded. Patients receiving systemic treatment with strong CYP1A2 inhibitors or strong inhibitors/inducers of CYP3A within 14 days were excluded. Other factors that precluded participation in the trial included uncontrolled cardiovascular conditions (including uncontrolled hypertension, uncontrolled cardiac arrhythmias, symptomatic congestive heart failure, unstable angina, or myocardial infarction within the past 6 months), known human immunodeficiency virus infection, known hepatitis B surface antigen-positive status, or known or suspected active hepatitis C infection, and known allergy to any of the study medications, their analogs, or excipients in the various formulations. Other comorbidities or severe pre-existing illness that in the treating physician’s opinion could interfere with oral absorption and/or tolerance of ixazomib citrate excluded patients from participation. Patients were characterized as having high-risk genetics if they had one of the following abnormalities on FISH testing: t(4;14), t(14;16), t(14;20) or del17p.

### Drug administration

All patients received ixazomib orally at a dose of 4 mg on days 1, 8, and 15 of a 28-day cycle along with dexamethasone at a dose of 40 mg orally was given on days 1, 8, 15, and 22 of the 28-day cycle. Dose modifications were made for ixazomib related toxicities with successive reductions in its dose to 3, 2.3, 2.3 mg every other week followed by discontinuation if the 2.3 mg dose every other week was not tolerated. Two doses of cyclophosphamide were evaluated in the phase 1 portion, 300 or 400 mg/m^2^, given weekly (days 1, 8, 15 and 22) during the 28-day cycle. Cyclophosphamide was progressively dose reduced to 300, 200 and 100 mg/m^2^ before discontinuation. Dexamethasone was decreased to 20, 12 and 4 mg prior to discontinuation. Prophylactic anti-emetics were recommended prior to each dose of ixazomib. Prophylactic anti-diarrheals were not used; however, the administration of anti-diarrheals was allowed after infectious causes were excluded. Topical steroids and other symptomatic measures were permitted for management of any skin rash. All patients were allowed to receive bisphosphonates and other supportive care as needed. Growth factor support for neutropenia was allowed beyond the first cycle of treatment in the phase 1 portion.

Induction therapy was defined as 12 cycles of the three-drug combination, after which patients could stay on single agent ixazomib at the last tolerated dose during induction phase, until disease progression, patient choice or unacceptable toxicity. Interruption of treatment for stem cell collection was allowed after completion of three cycles in patients who were transplant eligible and wanted to consider that option in the future. The choice of mobilization regimen was not specified by protocol. Patients went off study if at least a minor response (MR) was not seen after 3 cycles and at least a PR was not seen after 6 cycles, where the responses did not need to be confirmed.

### Assessments

Adverse events (AEs) were graded using the National Cancer Institute’s Common Terminology Criteria for AEs, version 4.0. Myeloma disease response was done in accordance with the International Myeloma Working Group uniform criteria, incorporating the additional category of MR. All response categories required confirmation of the required tests with the exception of the bone marrow used for CR determination. At any point in treatment, patients suspected of PD had response assessments repeated to confirm disease progression. If patients did not have confirmation and went on to another treatment, they were classified as having progressive disease.

#### Pharmacokinetic studies

Peripheral blood samples were collected during cycle 1 at 1 h pre-dose on days 1, 8, 15 and any time day on 22, and cycle 2 day 1. In addition, a 4-hour post-dose sample was obtained on cycle 1, day 1. Blood was collected using K2EDTA as anticoagulant and centrifuged for 10 min at 1006 × g at 4 °C in a refrigerated centrifuge within 10 min of sample collection. The plasma was separated and placed into vials containing lyophilized citric acid. The analyses were performed by Tandem Labs, West Trenton, NJ.

#### Neurotoxicity questionnaires

The FACT/GOG neurotoxicity questionnaires were completed by patients at baseline, after each cycle for the first 4 cycles, and then every three cycles thereafter.

### Statistical analyses

The primary end point of the phase 1 portion of this trial was to assess the maximum tolerated dose (MTD). The phase 1 portion of the study was expected to require a minimum of 6 and a maximum of 12 evaluable patients. For the phase 2 portion of this trial, the primary end point was the rate of complete or very good partial response. A success was defined as a CR or VGPR noted as the objective status on two consecutive evaluations. All patients meeting the eligibility criteria who signed a consent form and received at least one dose of the drug were evaluable for response, with the exception of patients who were determined to be a major treatment violation. The sample size for the phase 2 portion of the study was calculated using a one-stage binomial design. The 6 patients treated at the MTD in the phase 1 portion were also included in the phase 2 portion for overall sample size estimation. A maximum of 35 additional evaluable patients were planned to be accrued at the MTD dose level for a maximum of 41 evaluable patients in the phase 2 portion of this study. An additional 4 patients were accrued to account for ineligibility, cancellation, major treatment violation, or other reasons. With 41 evaluable patients, the study provided 90% power to test the null hypothesis that the ≥ VGPR rate is at most 40% vs. the alternative hypothesis that the ≥ VGPR rate is at least 60%, with a 1-sided significance level of *α* = 0.10. For toxicity assessment, all patients who received at least 1 dose of study drug were included in the analysis. Overall survival (OS) was defined as the time from study entry to death due to any cause. Progression-free survival (PFS) was defined as the time from study entry to the earliest date of documentation of disease progression or death due to any cause. Duration of response (DOR) was defined as the time from first evidence of a response until the time of disease progression. Time-to-event measures were estimated using the Kaplan–Meier method.

## Results

### Patients

Fifty-one patients were enrolled; including 10 patients in phase 1 (3 at 300 mg/m^2^ and 7 at 400 mg/m^2^) and 41 patients in phase 2. Two patients from the phase 1 portion and one patient from phase 2 were excluded from all analysis due to ineligibility. Data was frozen as of May 2017. The median age for the 48 evaluable patients was 64.5 years (range: 41–88) and 52% were male. The baseline characteristics at study entry for the entire study (*n* = 48) and for those in the phase 2 dosing (*n* = 45) are described in Table 1. Across the entire study, at the time of data cutoff, 11 (23%) patients had progressed and 46 (96%) were alive, with a median follow-up of 25.6 months (range: 12.3–44.6). Eight patients remain on therapy; reasons for drug discontinuation (*N*, %) were alternative treatment including transplant (19, 48%), disease progression (10, 25%), patient refusal (3, 8%), adverse event (3, 8%) and lack of pre-specified response threshold (5, 10%). Patient disposition by study arm is outlined in Supplementary Figure 1.

**Table 1:** Baseline characteristics

	**All patients**	**Phase 2 dose**
	**(*N*** = 48)	**(*N*** = 45)
Age: Median (Range)	64.5 (41.0–88.0)	64.0 (41.0–88.0)
Gender: Male	25 (52.1%)	24 (53.3%)
ECOG performance score		
0	25 (52.1%)	23 (51.1%)
1	19 (39.6%)	18 (40.0%)
2	4 (8.3%)	4 (8.9%)
mSMART risk		
Standard risk	34 (70.8%)	31 (68.9%)
High or intermediate	14 (29.2%)	14 (31.1%)
Abnormal metaphase cytogenetics	12 (25.0%)	12 (26.7%)
RISS		
Stage 1	15 (34.1%)	14 (34.1%)
Stage 2	27 (61.4%)	25 (61.0%)
Stage 3	2 (4.5%)	2 (4.9%)
Patients with stem cells collected	28 (58.4%)	28 (62.2%)

### Response to therapy and survival

**Fig. 1:**
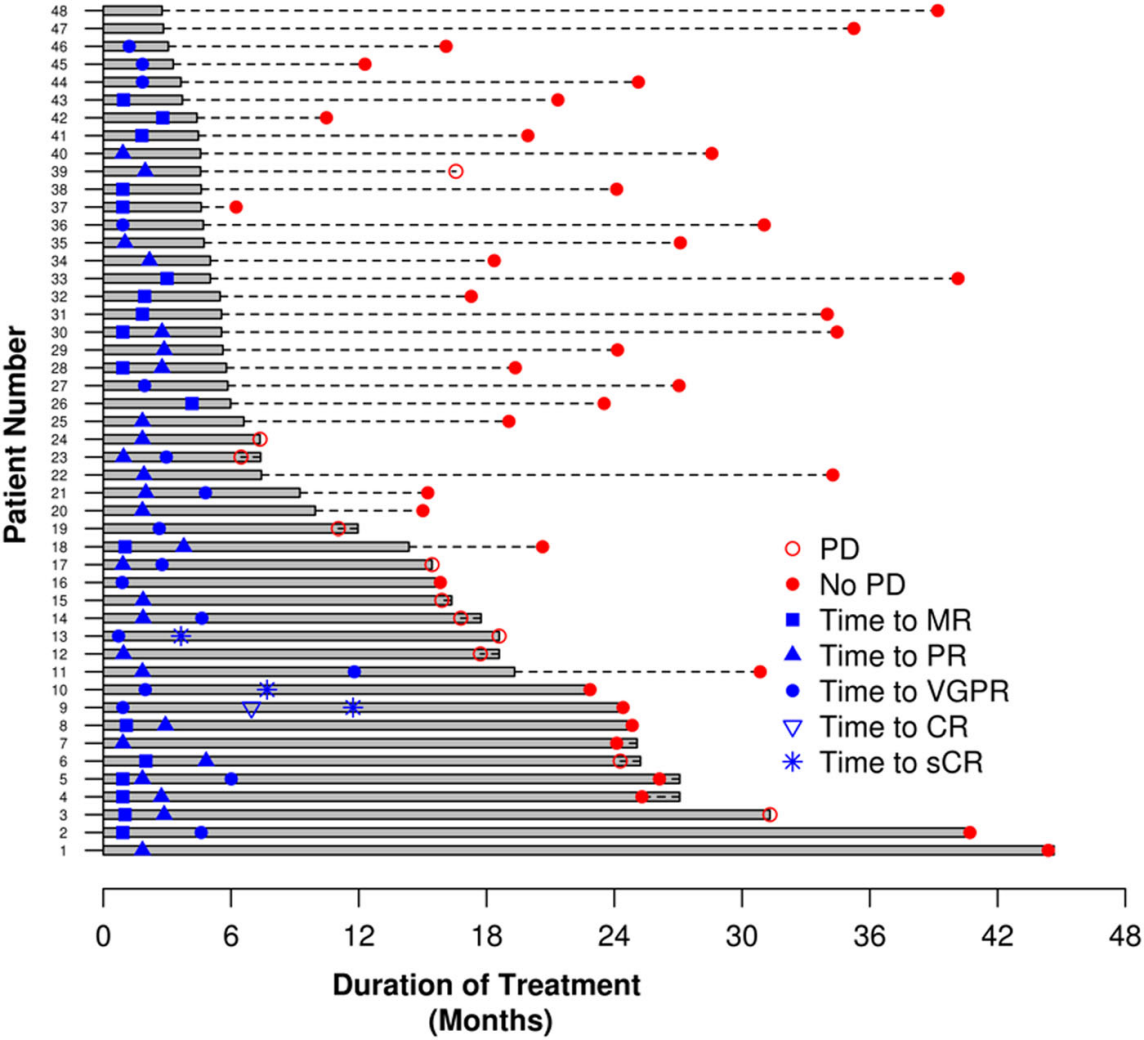
: Figure shows a swim plot depicting time to response per patient

Overall, 37 (77%, 95% CI: 63, 88) patients achieved a confirmed partial response or better across the entire trial, with 35% (95% CI: 22, 50) achieving a VGPR or better. The response rates for the entire study as well as the 45 patients who were enrolled at the MTD are shown in Table 2, including response rates grouped by FISH based risk status. There was increasing depth of response as patients continued to receive additional cycles of therapy as indicated by Fig. 1. Responses occurred rapidly, with the median time to response being 1.9 months (range: 0.9–4.8). Among the 48 patients, the response rate at the end of 4 cycles was 71%. Per protocol, 5 patients went off study for lack of an adequate response by pre-specified time point: MR not seen after 3 cycles (*n* = 1) and PR not seen after 6 cycles (*n* = 4).

**Table 2:** Efficacy outcomes and follow-up status

	**All patients**	**Phase 2 dose**
	**(N** **=** **48)**	**(N** **=** **45)**
Overall response rate	77% (95%CI: 63, 88)	78% (95%CI: 63, 89)
≥VGPR Response Rate	35% (95%CI: 22, 50)	38% (95%CI: 24, 53)
sCR	3	3
CR	0	0
VGPR	14	14
PR	20	18
MR	9	8
SD	2	2
Overall Response Rate by FISH		
High risk	88% (95%CI: 47, 100)	88% (95%CI: 47, 100)
Standard risk	75% (95%CI: 59, 87)	76% (95%CI: 59, 88)
Median overall survival^a^	NA	NA
12 Months	100%	100%
Median progression-free survival^a^	NA (95%CI: 31.3, NA)	NA (95%CI: 31.3, NA)
Median duration of response^a^	NA (95%CI: 30.3, NA)	NA (95%CI: 30.3, NA)
Median time to response	1.9 mos (range: 0.9–4.8)	1.9 mos (range: 0.9–4.8)
Patients with progression	11 (22.9%)	11 (24.4%)
Patients alive	46 (95.8%)	43 (95.6%)
Median follow-up (alive patients)	25.6 mos (range: 12.3–44.6)	25.1 mos (range: 12.3–40.7)
Last cycle administered	7 (range: 3–46)	7 (range: 3–44)
On treatment	8	7
Reason For ending treatment		
Refused further treatment	3 (7.5%)	2 (5.3%)
Adverse event	3 (7.5%)	3 (7.9%)
Disease progression	10 (25.0%)	10 (26.3%)
Alternate treatment	19 (47.5%)	18 (47.4%)
Lack of response	5 (10.4%)	5 (11.1%)

*CI* confidence interval, *mo* month, *NA* not attained

^a^ Kaplan–Meier

**Fig. 2:**
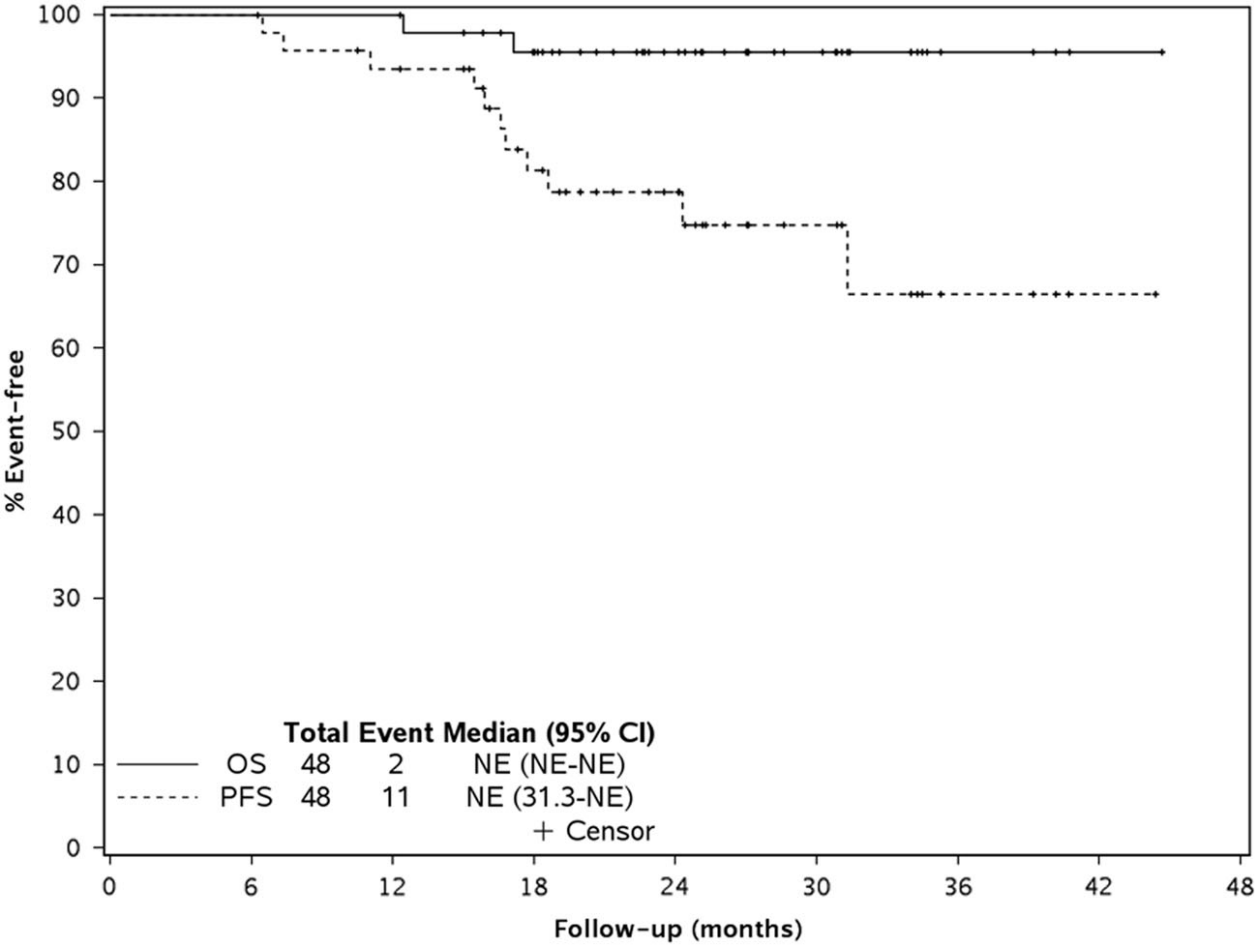
: Figure shows the overall survival (OS) and progression-free survival (PFS)

The median progression-free survival for the entire study population was not reached (NR) (95% CI: 31.3, NR); 18-month progression-free survival rate was 81% (95% CI: 70, 94). Median overall survival for the entire group was not reached; 18-month rate was 96% (95% CI: 90, 100) and 1-year overall survival was 100% (Table 2, Fig. 2). Across the entire study, the median PFS was not reached for patients with mSMART high/ intermediate risk (95%CI: 15.4, NR) or for those with standard risk genetics (95% CI: 31.3, NR). The median DOR among the 37 patients with a partial response or better was not reached (95% CI: 22.3, NR) with 26 responders were still progression-free after a median of 23.7 months (range: 11.3–42.5).

### Dose intensity and adverse events

Patients received a median of 7 cycles of therapy (range: 3–46) across the trial; 44 and 23 patients received at least 4 and 8 cycles respectively and 18 patients remained on trial for more than 12 cycles. The total number of cycles delivered across the entire trial and the number of patients requiring dose reductions for each drug is given in Table 3. The ixazomib dose delivered by cycle for the first 12 cycles, reflecting the need for dose reductions, is as shown in supplementary figure 2. Overall, 40 patients have gone off study, the most common reason being alternate therapy, which was a stem cell transplant in 12 patients. The reasons for discontinuation of protocol treatment are as shown in Table 2. There were no treatment related deaths.

**Table 3:** Treatment administration

	**All patients**	**Phase 2 dose**
	**(N** **=** **48)**	**(N** **=** **45)**
Ixazomib		
Number of cycles	620	561
Median dose (mg, Range)	12 (3–120)	12 (3–120)
Number of patients with dose reductions	6 (13%)	6 (13%)
Total number of dose reductions	8	8
Cyclophosphamide		
Number of cycles	382	357
Median dose (mg, Range)	2800 (0–5000)	3000 (0–5000)
Number of patients with dose reductions	16 (33%)	16 (36%)
Total number of dose reductions	24	24
Dexamethasone		
Number of cycles	386	361
Median dose (mg, range)	160 (0–160)	160 (0–160)
Number of patients with dose reductions	20 (42%)	18 (40%)
Total number of dose reductions	29	26
Treatment delays		
Number of patients	14 (25%)	12 (27%)
Total number of delays	19	15

**Fig. 3:**
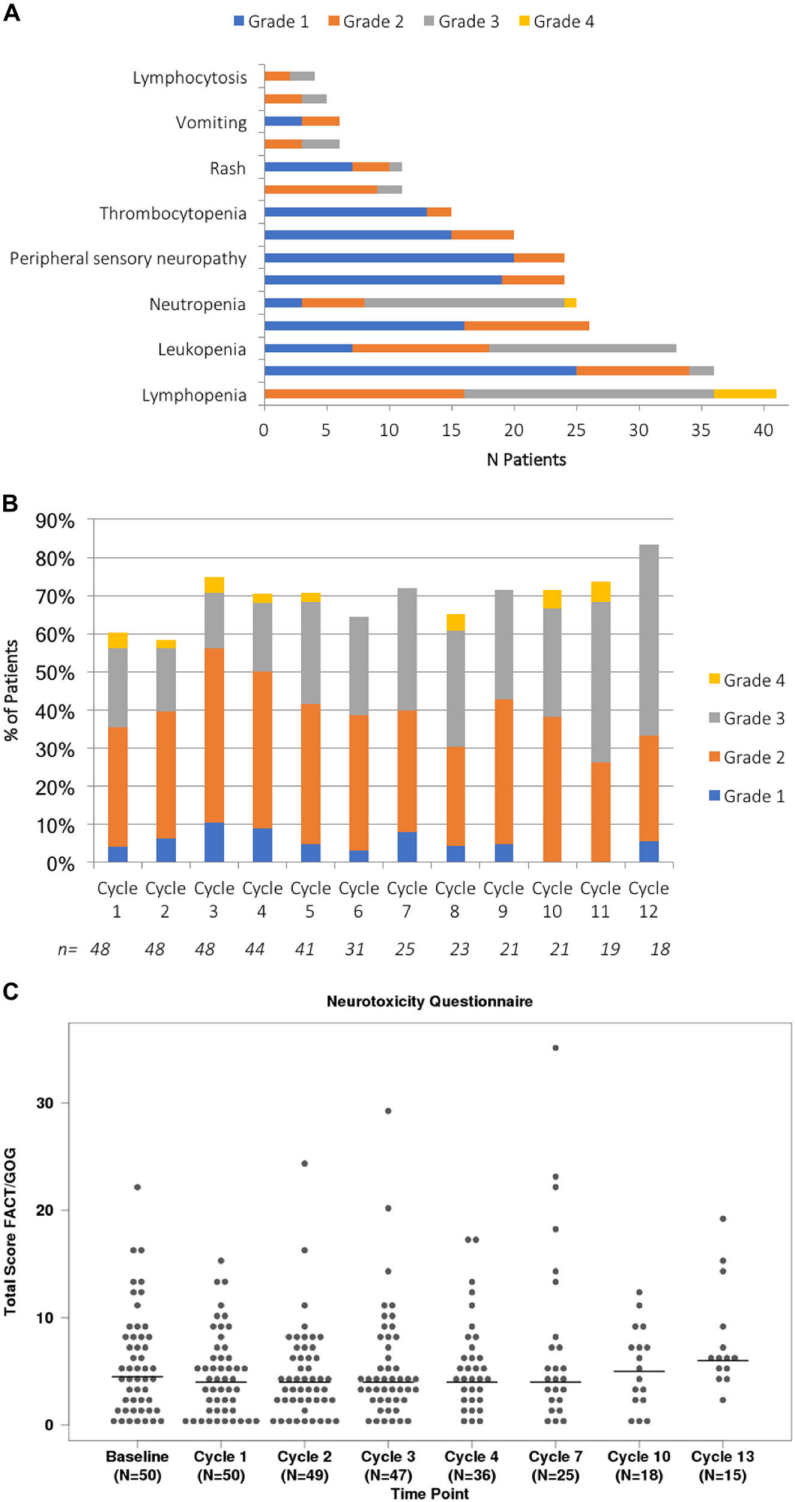
: **a** Provides the distribution of all grades of toxicities considered at least possibly related to the drug administration. **b** Shows the incidence of hematological toxicity across individual cycles, highlighting lack of any cumulative hematological toxicity. **c** Shows the distribution of Neurotoxicity data by cycle. The horizontal bars are the median total FACT/GOG score for each cycle

An AE of any grade, that was considered at least possibly related, was reported in 100% of the patients. There were no dose-limiting toxicities among the 8 patients enrolled in the dose escalation phase. A grade 3 or 4 adverse event considered at least possibly related to drug administration was seen in 42 (88%) patients (Table 4). The most common grade 2 or higher toxicities included lymphopenia, leukopenia, neutropenia, anemia, and nausea. Figure 3a provides the distribution of all grades of toxicities considered at least possibly related to drug administration. No cumulative hematological toxicity was observed across the entire trial as shown in Fig. 3b. Peripheral neuropathy at least possibly related to the drug was seen in 23 patients (grade 1), 5 patients (grade 2), and no patients experienced grade 3 or higher peripheral neuropathy. No cumulative neurotoxicity was seen as shown in Fig. 3c.

**Table 4:** Adverse events

	**Regardless of attribution**	**At least possibly Related**
Evaluable	48	48
Grade 3+	38 (79%)	36 (75%)
Grade 4+	6 (13%)	6 (13%)
Grade 5	0 (0%)	0 (0%)
Grade 3+ hematologic	34 (71%)	33 (69%)
Grade 4+ hematologic	6 (13%)	6 (13%)
Grade 3+ non-hematologic	13 (27%)	11 (23%)
Grade 4+ non-hematologic	2 (4%)	1 (2%)

### Stem cell collection and transplant outcomes

A total of 28 patients went on to stem cell collection while on study after a median of 5 cycles. The stem cell mobilization was performed with growth factor alone and plerixafor added in a risk-adapted manner or with cyclophosphamide priming. The median CD34 cell count was 9.2 million cells/kg (range: 4–51); no patient failed to collect at least 2.5 million CD34 cells/kg. Of the 28 patients with stem cell collection, 19 patients proceeded to an autologous stem cell transplant, after a median of 5 (range: 3–20) cycles of ICD. The response rate at the time of SCT, among those going to a transplant was 68%, including 4 patients with a VGPR or better.

### Ixazomib pharmacokinetics

**Fig. 4:**
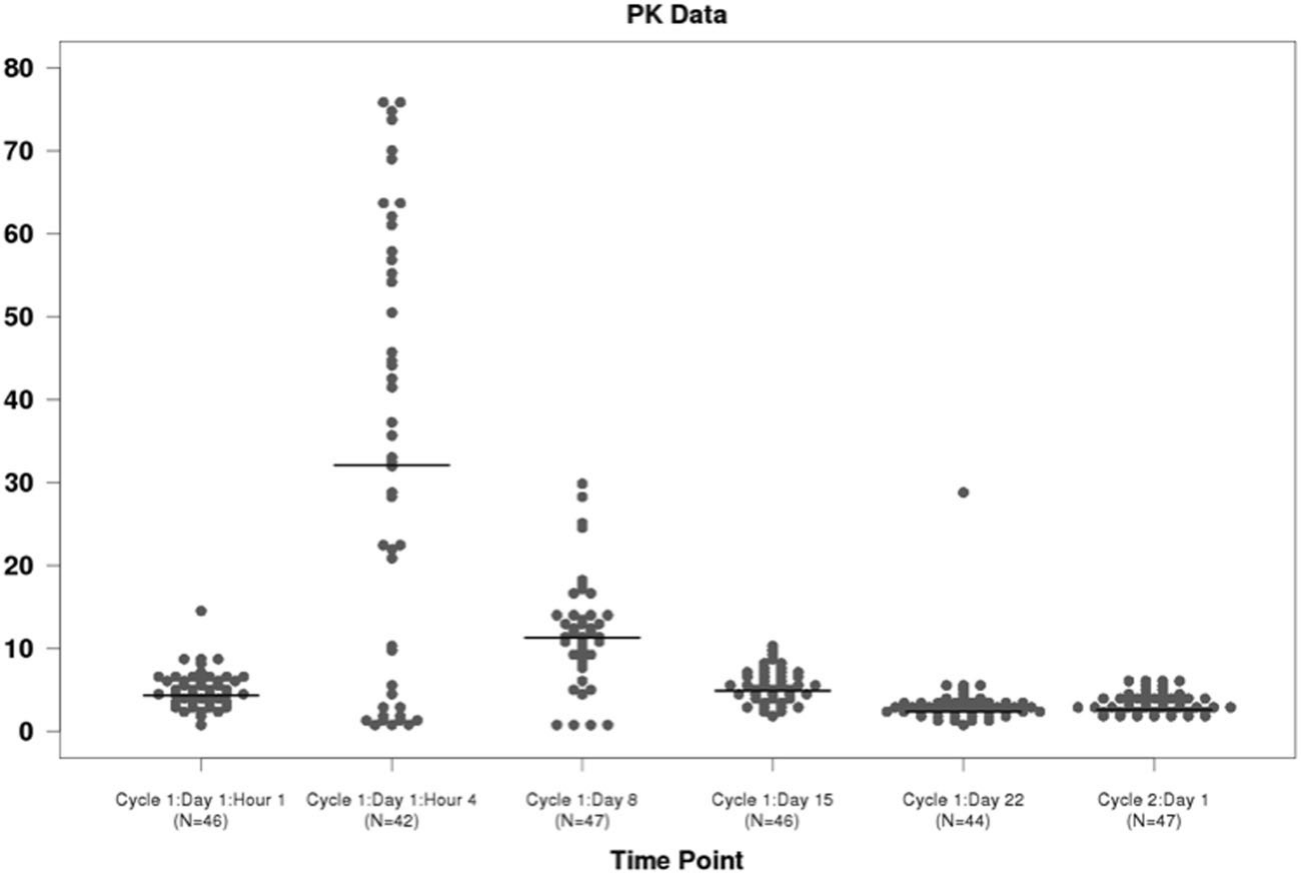
: Figure shows the individual ixazomib plasma concentration data by time point. The horizontal bars are the median concentration at each time point

The pharmacokinetic studies demonstrate a profile that is similar to what has been observed with single agent ixazomib as well as ixazomib used in combination with lenalidomide and dexamethasone in previous studies (Fig. 4).

## Discussion

The current study, to our knowledge, is the first to report on the combination of ixazomib, cyclophosphamide and dexamethasone in patients with newly diagnosed myeloma. Given the activity and long-term tolerability of ixazomib used in combination with lenalidomide and dexamethasone in patients with newly diagnosed as well as relapsed myeloma, other potential combinations are of great interest. In particular, the combination with cyclophosphamide required evaluation given the efficacy observed with bortezomib, cyclophosphamide and dexamethasone (VCd) as induction therapy in patients with newly diagnosed MM. Given the low risk of neurotoxicity with ixazomib, it offers the possibility of an all oral regimen, that can be tolerated for long periods of time, which is less expensive compared with the lenalidomide combination.

The VCd combination has been studied with different dosing schedules and intensity of all the three drugs. The initial combination by Reeder et al used twice weekly bortezomib along with intense dexamethasone dosing, and resulted in high response rates, but more toxicity related to the intense steroid schedule^31^. This was modified and a second cohort was studied with less intense steroids dosing and higher bortezomib dose (1.5 mg/m2) given once weekly, with comparable response rates^32^. The overall response rate was nearly 90% among the entire study cohort. The EVOLUTION trial also examined VCd with twice weekly bortezomib and with two different schedules of cyclophosphamide, one given 2 of the 3 weeks and another (modified VCd) with cyclophosphamide every week. The efficacy seen here is comparable with that observed with the VCD regimen in that trial: 75% for the VCd, though a 100% response was seen among the 17 patients treated with modified VCd. One of the largest trials with VCd was the phase 3 French trial comparing VCd with VTd, where bortezomib was given twice weekly with 500 mg/m^2^ of cyclophosphamide and pulsed dose dexamethasone. The overall response rate was 83% in an intent to treat analysis among the VCd group, albeit with 4 cycles of therapy as this trial was intended to compare the two regimens for induction therapy prior to transplant. The current regimen is less intense compared to the VCd regimens studied, with weekly PI, weekly Dexamethasone and lower dose of cyclophosphamide (400 mg/m^2^). Also, the current trial included transplant eligible and non-eligible patients with patients up to 88 years of age and despite this the regimen was well tolerated with good efficacy. It is also important to compare and contrast the results with that seen with the VTd combination. This triplet has been extensively used in Europe and Asian countries, particularly given the cost and the access to lenalidomide. In the phase 3 trial, the response rates were significantly higher for the VTd regimen, making it an effective induction therapy. However, the neurotoxicity was considerable making it less acceptable to the older patient population and in those prolonged therpay is being considered.

It is relevant to compare and contrast the results with the lenalidomide combinations of bortezomib and ixazomib, given the excellent results seen with VRd in the phase III SWOG trial, which has resulted in VRd being the most commonly used induction therapy in the US. The results of the SWOG trial (S0777) with the VRd combination is most comparable to the current trial in terms of the patient population enrolled, where the overall response with VRd was 81.5% with a VGPR or better of 42%. The combination of ixazomib, lenalidomide and dexamethasone (IRd) is a well-tolerated regimen in patients with newly diagnosed myeloma, both transplant eligible and ineligible. In a phase 2 trial, IRd was associated with an overall best response rate of 92% with increasing depth of response seen with longer duration of therapy. While the response rates are difficult to be compared directly between two phase-2 trials, ICd appears to be an effective regimen in the setting of newly diagnosed myeloma, although possibly with lower response rates than was observed with the lenalidomide combination. A direct comparison of VCd and VRd or ICd and IRd are not available from a clinical trial setting. In a retrospective study of the two regimens, the efficacy appeared to be comparable between the VCd and VRd regimens, but no similar data exist as of now for ICd and IRd^33^. As with the bortezomib and lenalidomide combinations, longer duration of therapy translated into a deeper response and the lack of peripheral neuropathy can potentially allow for longer therapy.

The toxicity profile of the regimen has been very similar to that seen with the VCd regimens, with the exception of peripheral neuropathy. No grade 3 or higher PN was seen in this cohort, compared to 12–17% grade 3 and higher PN seen with the bortezomib combinations. We undertook a detailed evaluation of peripheral neuropathy over time using prospectively administered patient questionnaires and demonstrated a lack of any cumulative neurotoxicity. As with other ixazomib combinations, we did observe gastrointestinal toxicity, particularly nausea, but this was manageable with dose modifications and supportive care measures. Hematological toxicity remains the most common category of adverse events observed but was similar in spectrum as has been seen with the VCd and IRd combinations. No cumulative hematological toxicity was observed across the multiple cycles of the combination among those who continued on therapy. For patients, who plan to collect stem cells, no adverse effect was seen on the ability to collect stem cells. This is consistent with what has been observed with VCd or IRd.

In conclusion, the combination of ixazomib, cyclophosphamide and dexamethasone is a well-tolerated and effective, all oral combination that can be used for initial therapy of newly diagnosed myeloma. The efficacy is comparable to that seen with bortezomib-based regimens with significantly lower neurotoxicity. The convenience and tolerability makes this regimen particularly relevant for the older, non-transplant eligible patient in whom longer term therapy is planned. In comparison to the lenalidomide based combination, cyclophosphamide offers a lower cost option without compromising the efficacy and the ability to move ahead with stem cell collection and autologous transplant, if eligible and desired. Additionally, ICd can serve as a foundation to which monoclonal antibodies can be combined to further improve efficacy without the cost of three novel agents used simultaneously.

## Electronic supplementary material

Supplementary Figure Legends

Supplementary Figure 1

Supplementary Figure 2
