# Supplementary material for: Phase 1/2 trial of ixazomib, cyclophosphamide and dexamethasone in patients with previously untreated symptomatic multiple myeloma
Source: Blood Cancer J. 2018 Jul 30;8(8):70. doi: 10.1038/s41408-018-0106-3 (PMC6066484; doi:10.1038/s41408-018-0106-3)
Supplement: Supplementary file 1 — Supplementary Figure Legends [file 41408_2018_106_MOESM1_ESM.docx]

**Supplementary Figure Legends**

**Supplementary figure 1**: Figure shows the disposition of patients over the trial duration.

**Supplementary figure 2:** The ixazomib dose delivered by cycle for the first 12 cycles.
