# Supplementary figures and images for: Phase 1/2 trial of ixazomib, cyclophosphamide and dexamethasone in patients with previously untreated symptomatic multiple myeloma
Source: Blood Cancer J. 2018 Jul 30;8(8):70. doi: 10.1038/s41408-018-0106-3 (PMC6066484; doi:10.1038/s41408-018-0106-3)

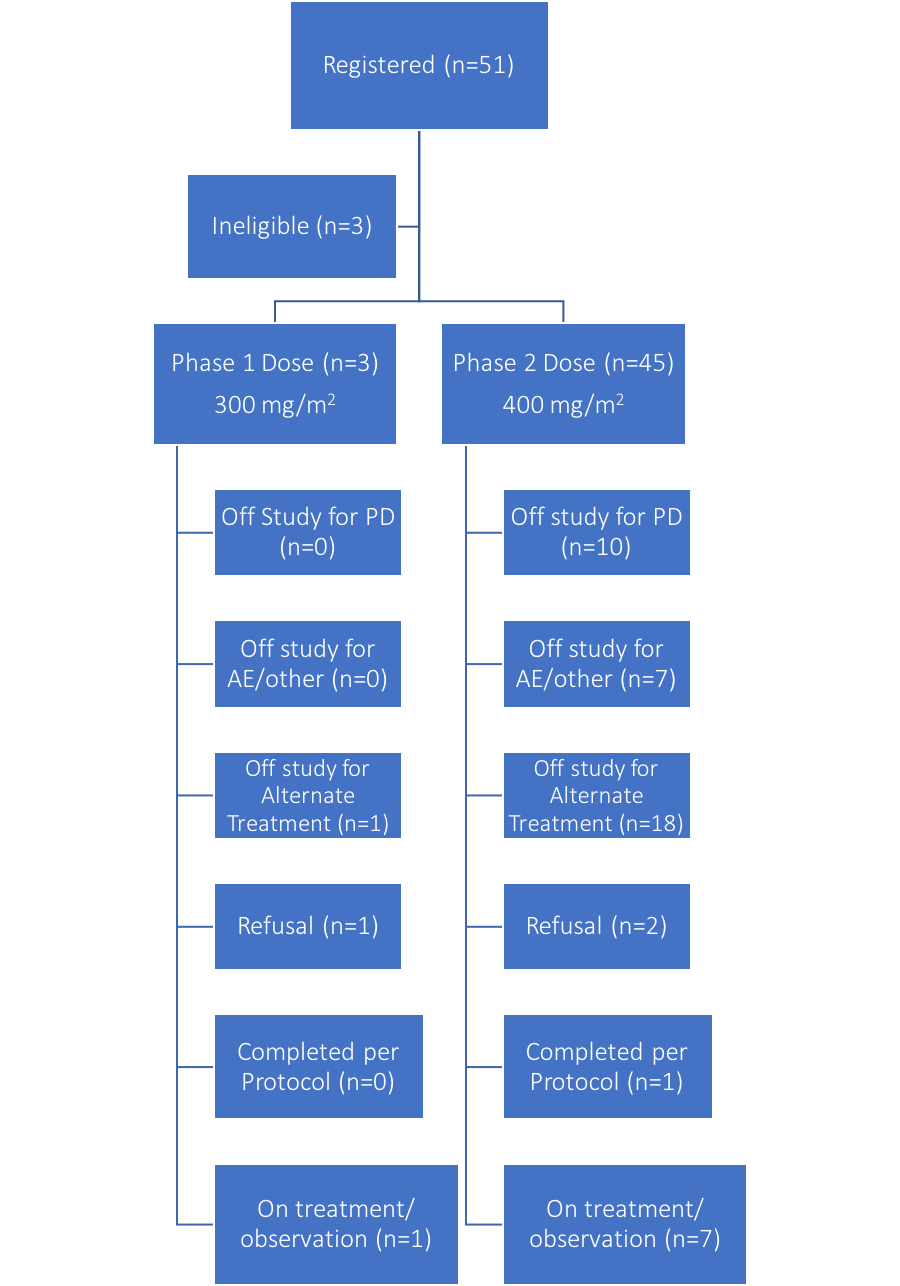

Supplement: Supplementary file 2 — Supplementary Figure 1 [file 41408_2018_106_MOESM2_ESM.png]

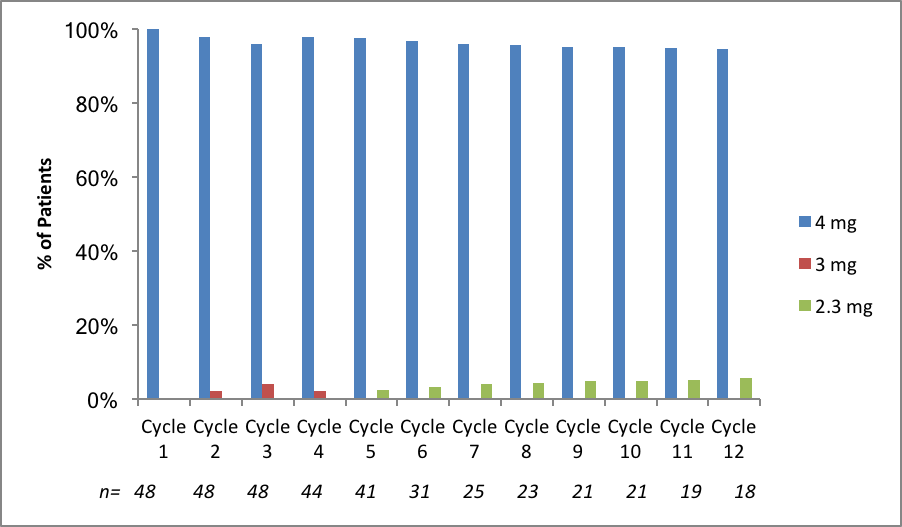

Supplement: Supplementary file 3 — Supplementary Figure 2 [file 41408_2018_106_MOESM3_ESM.png]
